# A Multi-Sensor RSS Spatial Sensing-Based Robust Stochastic Optimization Algorithm for Enhanced Wireless Tethering

**DOI:** 10.3390/s141223970

**Published:** 2014-12-12

**Authors:** Ramviyas Parasuraman, Thomas Fabry, Luca Molinari, Keith Kershaw, Mario Di Castro, Alessandro Masi, Manuel Ferre

**Affiliations:** 1 European Organization for Nuclear Research (CERN), Geneva 1211, Switzerland; E-Mails: thomas.fabry@cern.ch (T.F.); luca.molinari@cern.ch (L.M.); keith.kershaw@cern.ch (K.K.); mario.di.castro@cern.ch (M.D.C.); alessandro.masi@cern.ch (A.M.); 2 CAR UPM-CSIC, Universidad Politécnica de Madrid, Madrid 28006, Spain; E-Mail: m.ferre@upm.es

**Keywords:** wireless tethering, relay, wireless nodes, mobile robots, multi-sensor sampling, RSS, receiver spatial sampling

## Abstract

The reliability of wireless communication in a network of mobile wireless robot nodes depends on the received radio signal strength (RSS). When the robot nodes are deployed in hostile environments with ionizing radiations (such as in some scientific facilities), there is a possibility that some electronic components may fail randomly (due to radiation effects), which causes problems in wireless connectivity. The objective of this paper is to maximize robot mission capabilities by maximizing the wireless network capacity and to reduce the risk of communication failure. Thus, in this paper, we consider a multi-node wireless tethering structure called the “server-relay-client” framework that uses (multiple) relay nodes in between a server and a client node. We propose a robust stochastic optimization (RSO) algorithm using a multi-sensor-based RSS sampling method at the relay nodes to efficiently improve and balance the RSS between the source and client nodes to improve the network capacity and to provide redundant networking abilities. We use pre-processing techniques, such as exponential moving averaging and spatial averaging filters on the RSS data for smoothing. We apply a receiver spatial diversity concept and employ a position controller on the relay node using a stochastic gradient ascent method for self-positioning the relay node to achieve the RSS balancing task. The effectiveness of the proposed solution is validated by extensive simulations and field experiments in CERN facilities. For the field trials, we used a youBot mobile robot platform as the relay node, and two stand-alone Raspberry Pi computers as the client and server nodes. The algorithm has been proven to be robust to noise in the radio signals and to work effectively even under non-line-of-sight conditions.

## 1. Introduction

Networks of mobile wireless robots are increasingly considered in applications in hazardous environments where humans cannot perform some tasks because of safety issues and challenges in the environments [1,2]. For instance, in underground scientific facilities [3], such as at CERN (the European Organization for Nuclear Research), mobile robot nodes could help in remote inspections and radiation surveys in different areas [4-8]. The use of wired communication in hostile environments has not proven reliable, with fiber-optic cables susceptible to tangling, breakage and being run over by the robots [2]. Wireless communication offers benefits over wired communication systems, such as low maintenance, higher robustness against failures stemming from physical damage, less manpower needed for managing the tethers and ease of mobility. Typical requirements for a wireless robot in hostile or underground environments are given below:

(1) the wireless robot should be able to travel distances longer than 200 m [6];
(2) the robot should communicate with the base station, in the possible presence of electromagnetic fields and/or ionizing radiation, and should be able to avoid wireless communication failures, radio interference, multipath and deep fading effects [3,9,10];
(3) the robot shall in no way interrupt the operation or damage any objects in the facilities where it is deployed.

**Figure 1.**
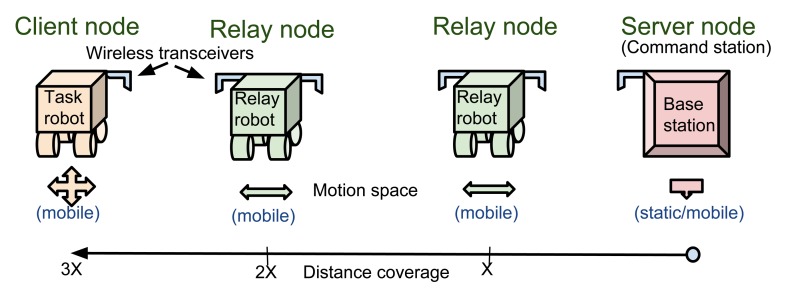
Server-relay-client (SRC) architectural view of this study.

To meet these requirements and, more specifically, to improve wireless communication performance in non-line-of-sight (NLOS) conditions and underground tunnels, the concept of wireless tethering using intermediate relay nodes (forming a network of mobile robotic nodes) has been suggested by previous researchers [9–13]. A commonly-used model for networked wireless nodes is the server-relay-client (SRC) framework, as shown in Figure 1. In addition to the need for relay nodes, there is also a need for redundancy features to improve reliability, avoid communication failure and enable recovery in the event of failure in the wireless network.

**Figure 2.**
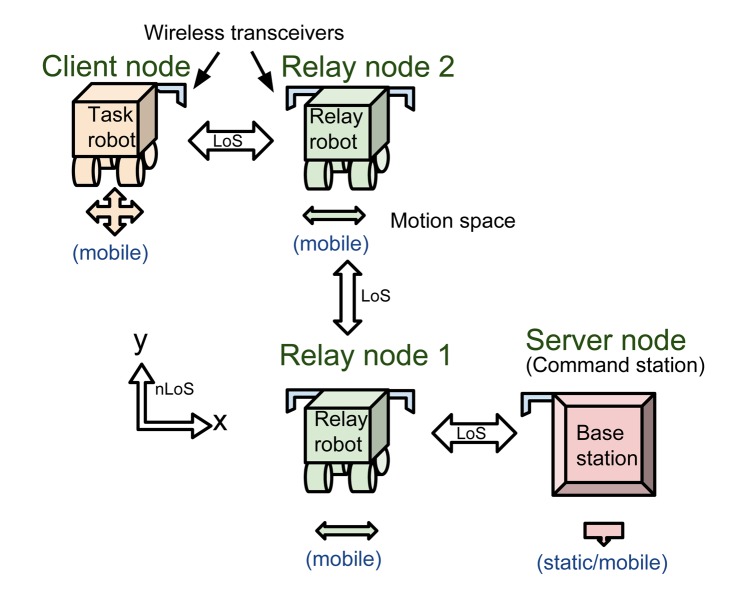
An example architectural view of the SRC networked wireless robot node system.

Figure 2 shows an example of using relay nodes for multi-hop wireless tethering from an operator command station to the client/task node in line-of-sight (LOS) or NLOS conditions. If the relay nodes are autonomous with decentralized operations, this allows the human operator to concentrate on controlling only the client node. An important intelligence feature necessary for the relay nodes is that they must be able to optimize their positions to maintain a stable, end-to-end communication link.

The wireless link quality and the network throughput have a strong correlation with the received radio signal strength (RSS) and the signal-to-noise ratio (SNR) [14,15]. It has been demonstrated in [16,17] that the network throughput can be improved by balancing the RSS (at the relay node) from the server and client nodes. As the RSS is proportional to the logarithmic distance between two wireless nodes in LOS conditions, the RSS at each side of the relay node can be balanced by optimizing its position. Moreover, continuously optimizing the relay node's position will enhance the wireless network performance as the dynamics in the environment (such as fading effects and movements of various objects) influence the RSS [18].

In our previous work [15], we exploited spatial diversity techniques to optimize the RSS at a mobile robot node. In this paper, we extend our previous work and propose an adaptive robust stochastic optimization (RSO) algorithm for autonomous wireless relay positioning using a spatially spread RSS sensing technique to dynamically optimize the RSS assuming an unknown, noisy radio environment. Redundancy in the network is shown to be improved by making use of the proposed method, so that the system can tolerate, to some extent, wireless device failures, for instance caused by single event effects (SEE) of ionizing radiation in the environment. The proposed algorithm is made robust to noise in the measurement and environment by using pre-processing filtering techniques. To summarize, the main objective in this work is to navigate the relay node to a position (between two nodes) where the RSS is at a local maximum or at least above the minimum threshold needed to maintain a strong connectivity.

We first review the available literature in relay robot position optimization methods for improving communication quality in Section 2 and highlight how the proposed method differs from previous approaches. Secondly, we present an overview of the radio signal propagation and wireless network capacity followed by formally stating the problems considered in this paper in Section 3. We then continue by proposing a robust stochastic optimization algorithm and discuss its implementation in detail in Section 4. The proposed solution is evaluated with simulation experiments presented in Section 5, after which we validate the results with field trials in Section 6. Finally, we conclude the paper and summarize the key values and advantages of the proposed method.

## 2. State of the Art

Networking of wireless mobile robot nodes has been well-studied in the literature [17–24]. In particular, the use of relay nodes with principal features, such as self-configuration, self-healing and wireless tethering, is exploited in [13,16,20,21]. For instance, Fink *et al.* [18] propose a global search-based method, adopting a stochastic model for mobility planning of a group of robotic nodes to increase robustness in wireless networking.

The role of the RSS has been significant in most of the research in wireless communication performance optimization. Much research has been done on using the RSS in multi-robot wireless tethering and wireless localization [19,22,23,25]. In [22], a data-driven probabilistic model is used for RSS-based localization and wireless tethering algorithms.

Radio source seeking techniques have been studied using RSS gradient [15,19] and angle of arrival methods [25]. Gradient-based methods have proven to be promising for improving the wireless connectivity of mobile nodes [15,17,19,26]. The authors in [17] used aerial robots as relay nodes and used antenna diversity for autonomously positioning the relay node using a gradient search technique, *i.e.*, sampling the RSS from multiple antennas (in different directions) that are connected to a receiver and obtaining the direction of the highest RSS. Rizzo *et al.*. [27] also study the use of antenna spatial diversity in building wireless coverage maps and using it for optimal deployment of a wireless network. We have previously demonstrated in [15] that the temporal influence in RSS measurements cannot be neglected, and therefore, using time-independent approaches could boost the spatial diversity performance (in comparison to the time-dependent approaches used in [17,27]).

Most of the research solutions possess drawbacks, such as the use of customized hardware with changes at the physical layer in the network stack of the wireless router (for instance, using a motorized base for rotating directional antennas). The dependency on customized hardware design could be avoided by using algorithms acting directly in the application layer that can easily be integrated and shared. To the best of the authors knowledge, previous research had not utilized multiple receivers (for redundancy and spatial RSS sensing) and time-independent methods for improving the RSS [15].

In this paper, we borrow the concepts from [16,17,23,27] while intending to remove the hurdles and drawbacks observed in these state-of-the-art methods. The proposed method is novel in the following way compared to the state of the art (which also shows how we depart from others): no prior knowledge or model of the radio environment is required; a combination of spatial and temporal pre-processing techniques is used to deal with the measurement noise and stochastic nature of the RSS and to mitigate fading effects; multiple receivers spatially distributed on-board the relay node are used instead of using multiple antennas connected to the same receiver, therefore avoiding the need to switch between different antennas connected to the same receiver and also making the algorithm applicable directly at the application layer. We evaluate our proposed algorithm with simulations and experiments using a position controller on a robot that can work under both LOS and NLOS conditions and even when the RSS gradient measurements are noisy.

It has been shown in [28] that using multiple sensors improves redundancy and reduces the data loss rate. It is worth noting that the application of interest in [28] is a static bio-medical data collection system, and all of the redundant devices are widely spread, whereas in our method, all of the redundant sensors are placed in one moving (relay) node. Nevertheless, the redundancy advantage can still be proven, as shown in [27], that by using a multi-antenna (in our case, multi-sensor) spatial RSS sampling method, wireless network capacity could be improved.

The main contributions in this paper are listed below:

(1) A decentralized stochastic optimization algorithm (robust to measurement noise) employing a gradient ascent method that can be applied on the relay node using multi-sensor spatial RSS sampling is proposed to find the optimal position of the relay node between two wireless nodes.
(2) The proposed method is validated using simulations and field experiments considering performance metrics, such as the temporal and spatial performance of the relay node, accuracy and success rate in reaching the optimal position. The proposed algorithm is compared with the state-of-the-art methods at relevant places in this paper.

The proposed work is a step towards intelligent decentralized relay nodes that uses node mobility to improve connectivity in wireless tethering applications [16,17,19,20,22].

## 3. Background

### 3.1. Radio Signal Propagation

A commonly-used model for radio signal propagation as a function of distance and time is the log-normal shadowing model (LNSM), as shown in the Equation (1). The variation of the RSS in space and time is partly a deterministic and partly a stochastic process [29], because the signal propagates through a multipath fading channel (radio signals from the transmitter arriving at the receiver through multiple paths). The attenuation in the power of the radio signal is defined as the path loss *PL* and is caused by many factors, such as distance (free space loss) and multipath propagation effects. In particular, all walls, ceilings and other objects that affect the propagation of radio waves will directly influence the signal strength and the directions from which the radio signals are received. The loss in the RSS can be regarded as a sum of deterministic effects (modeled using a logarithmic function) and stochastic effects (modeled using random Gaussian variables [30]), as shown in the equation below:

(1) $$\begin{array}{ll} PL_{d}=\underset{\text{Deterministic effect}}{\underset{︸}{PL_{d_{0}}+10\cdot \eta \log_{10}(\frac{d}{d_{0}})}}+\underset{\text{Stochastic effect}}{\underset{︸}{\chi_{\sigma }}} & \left[\text{dB}\right] \end{array}$$

where $\left\|d\right\|=\sqrt{{\left(x_{t}-x_{r}\right)}^{2}+{\left(y_{t}-y_{r}\right)}^{2}}$is the Euclidean (*l*^2^-norm) distance between the transmitter at *p_t_* = (*x_t_*, *y_t_*) and the receiver at *p_r_* = (*x_r_*, *y_r_*), *PL_d_* is the loss (expressed in decibels) in the RSS at distance *d*, *PL_d_*_0_ is the path loss at a reference distance *d*_0_ and *η* ≥ 2 is an environment specific propagation constant. The decibel (dB) is a relative unit of power measurement, often used to quantify gain or loss in radio signal power (strength). $\chi_{\sigma }=\chi_{\sigma }+\overset{N}{\underset{i=1}{\text{∑}}}k_{i}L_{i}$is the stochastic part of the RSS and is generally termed as a wide-sense stationary (WSS) process (the channel is time invariant). Here, the *χ_σ_* models the stochastic nature of the signal attenuation and can be represented using a zero-mean Gaussian random variable with a variance *σ*^2^, and *L_i_* is the loss due to walls and ceilings where *k_i_* is a parameter depending on the type of such obstruction. However (as we see later), this generalization is invalid when we include multipath fading effects because the multipath channel is time-variant [10]. Hence, we generalize the stochastic nature of the RSS variations with both shadowing (time-invariant) and multipath fading effects (time-variant).

The RSS at the receiver is the difference between the transmitted power *P_T_* and the path loss *PL_d_* over a distance *d*. Therefore, *RSS_d_* = *P_T_* − *PL_d_*. The path loss *PL_d_* is composed of three main components, as shown in Equation (2);

(2) $$\text{RSS}(d,t)=P_{T}-PL_{d}^{fs}-PL_{d}^{sh}-PL_{\left(d,t\right)}^{mp}=RSS_{d_{0}}-\underset{\text{Free}-\text{space path loss}}{\underset{︸}{10\eta \log_{10}(\frac{d}{d_{0}})}}-\underset{\text{Shadowing}}{\underset{︸}{\Psi_{dB}(d)}}-\underset{\text{Multipath}}{\underset{︸}{\Omega_{dB}(d,t).}}$$

Here, $PL_{d}^{fs}=PL_{d0}^{fs}+10\eta \log_{10}\left(\frac{d}{d_{0}}\right)$is the distance-dependent free-space path loss component due to environmental factors, such as the size and the shape of the environment. $PL_{d}^{sh}$is the shadow fading component of the path loss accounting for attenuation caused by objects (including walls and ceilings) in the environment. $PL_{\left(d,t\right)}^{mp}$is the multipath fading component accounting for attenuation caused by dynamics in the multipath channel.

Free-space path loss is a slow process (large scale), whereas the fading due to shadowing and multipath effects are relatively medium-scale and fast processes. The path loss in the RSS due to shadowing effects *PL^sh^* = *ψ_dB_* is represented using a zero-mean Gaussian distribution $\left(\psi \sim N(0,\sigma_{\psi }^{2})\right)$with a variance $\sigma_{\psi }^{2}$. The multipath fading effects in the received signal is a function of both space and time; *PL^mp^* = Ω*_dB_* and are modeled as a Gaussian random variable $\left(\Omega \sim N(0,\sigma_{\Omega }^{2})\right)$with zero-mean and a variance $\sigma_{\Omega }^{2}$. The probability density function (pdf) of the *PL^mp^* can be structured using Rician fading, Rayleigh fading or Nakagami-m fading models for LOS, NLOS or indoor distributions, respectively [30]. The parameters *η*, *σ_ψ_* and *σ*_Ω_ together define the radio environment [29].

A radio environment usually contains noise signals (*PN*) from other radio sources operating in the same frequency band or higher power signals operating at any radio frequency nearby the radio receivers. As the noise in the environment is a non-stationary process because of many factors, including the mobility of the node, it is highly unpredictable and cannot usually be modeled. In order to account for the noise, a widely-used measure is the signal-to-noise ratio (SNR), which assesses the received radio signal level with respect to the noise level in the surroundings. The SNR is defined as follows:

(3) $$\text{SNR}=\frac{RSS_{W}}{PN_{W}}=10^{\left(\frac{SNR_{\text{dBm}}}{10}\right)}\text{where},SNR_{\text{dBm}}=RSS_{\text{dBm}}-PN_{\text{dBm}}$$

The *RSS_W_* is the absolute power (expressed in W) and *RSS_dBm_* = 10 log(*RSS_mW_*) is the RSS value in decibels referenced to the RSS of one milliwatt of the absolute power. The concept of conversion from absolute power to dBm applies also to the SNR and the noise signal. While the RSS is an indicator of the intended signal's power, the SNR is an indicator of the intended signal power with respect to the noise power in the environment.

The RSS can be measured directly (in terms of dBm) in most of the commercial wireless devices using a metric called the radio signal strength indicator (RSSI) (even though the RSSI metric may report different measurements, such as the absolute signal power (dBm), a relative signal level (unit-less), the SNR (dBm) or the bit error rate (BER) (%), due to its vendor-specific nature, the wireless devices used in this paper report absolute signal power (dBm) in RSSI) [31]; however, this is not the case for SNR measurements. In some devices, the noise power level (which is usually inaccurate [31]) and RSS values are reported separately, and hence, the SNR can be calculated using both of these measurements. To avoid device dependency and to widen the applicability of our algorithm, we use the RSS measurements instead of the SNR measurements.

### 3.2. Wireless Network Characteristics

According to the Shannon–Hartley capacity theorem [32], in a wireless channel *c*, the maximum communication channel capacity *C_c_* and the data throughput *T_c_* is related to the SNR as follows:

(4) $$C_{c}=B\log_{2}(1+\text{SNR})=B\log_{2}(1+10^{\frac{RSS_{\text{dBm}}-PN_{\text{dBm}}}{10}})[\overset{\text{Mb}}{\;}/\text{s}]$$

(5) $$T_{c}=\alpha_{T}C_{c}[\overset{\text{Mb}}{\;}/\text{s}]$$

Here, *B* is the frequency bandwidth of the channel in MHz. The data throughput *T_c_* is a factor (by *α_T_*) of the maximum capacity *C_c_*, because the capacity is determined at the “physical” layer in the open systems interconnection (OSI) model [33], whereas the throughput is determined at the “data transport” layer, which includes the data link, network and physical layers in the OSI model. As our objective is to implement our solution at the application level, we use a metric called “goodput” *G_c_*, which is defined as the application-level data throughput. The goodput of a network can be modeled with Equation (6) and is measured right at the “application” layer (the top-most layer in the OSI model). The goodput is characterized as follows:

(6) $$G_{c}(\text{RSS})=\{\begin{array}{ll} \alpha_{G}T_{c}(\text{RSS}) & \mathrm{when}\text{RSS}\leq RSS_{\text{threshold}}\\ G_{\text{max}} & \mathrm{when}\text{RSS}\geq RSS_{\text{threshold}} \end{array}$$

**Figure 3.**
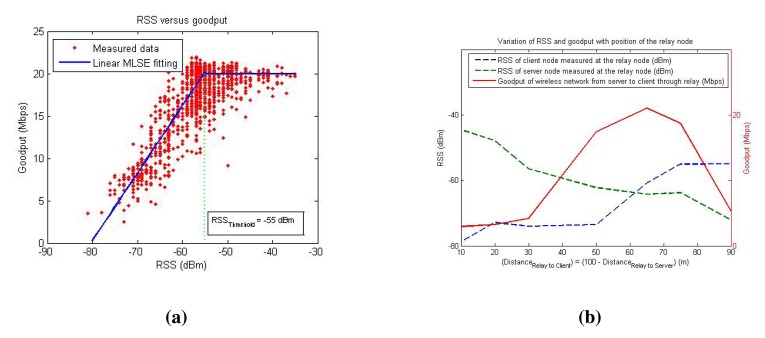
Relationship between the radio signal strength (RSS) and the goodput measurements. (**a**) Scatter plot depicting the measured goodput values against the RSS in various receivers at different time instants; (**b**) RSS and goodput values measured by a relay node at various positions and instants in a non-LOS (NLOS) condition.

*α_G_* is a parameter that determines the proportionality of the goodput with respect to the throughput. The model indicates that the goodput has a linear relationship with the maximum channel capacity *C_c_*. The goodput depends on the RSS and becomes saturated to a maximum level (*G_max_*) for a particular link when the RSS reaches an upper threshold *RSS*_threshold_ (which is different for different wireless device). This model is verified with the experimental data presented in Figure 3a, where the *RSS*_threshold_ is observed to be −55 dBm. The lower threshold for the RSS is normally the sensitivity (*R_S_*) of the wireless sensor, which in this case is −82 dBm (for a Zyxel NWD2105 device operating in the 2.4 GHz 801.11b band). Figure 3a also shows a linear fit for the RSS and goodput relation using a minimum least squares error (MLSE) technique. The goodput model considered in this work is a more practical model than the Shannon capacity model in Equation (5).

A network can make use of a relay node using either a relay-and-retransmit (RR) scheme or a flow-pipe (FP) scheme for data transport through the relay [16]. While the former is based on packet switching, the latter has the functionality of a radio-repeater. The total network goodput *G_t_* of a robotic network having *n* nodes (the first being the server node, the *n^th^* node being the client node and *n* − 2 relay nodes in between the server and the client nodes) is given by [34]:

(7) $$G_{t}=\{\begin{array}{ll} {\left[\underset{\begin{array}{c} i\in [1,n-1]\\ j=i+1 \end{array}}{\text{Σ}}\frac{1}{G_{ij}}\right]}^{-1}={\left[\underset{\begin{array}{c} i\in [1,n-1]\\ j=i+1 \end{array}}{\text{Σ}}\left(\frac{1}{\alpha_{T}\alpha_{G}B{\text{log}}_{2}(1+10\frac{RSS_{\text{dBm}}(i,j)-PN_{\text{dBm}}(i,j)}{10})}\right)\right]}^{-1} & \text{for the RR scheme}\\ \underset{\begin{array}{c} i\in [1,n-1]\\ j=i+1 \end{array}}{\min}\{G_{ij}\}=\underset{\begin{array}{c} i\in [1,n-1]\\ j=i+1 \end{array}}{\min}(\alpha_{T}\alpha_{G}B{\text{log}}_{2}(1+10\frac{RSS_{\text{dBm}}(i,j)-PN_{\text{dBm}}(i,j)}{10})) & \text{for the FP scheme} \end{array}$$

where *G_ij_*, and *RSS_dBm_*(*i*, *j*) represent the goodput of the link and the received signal strength (from node *j* to node *i*), respectively. In this work, we assume that we use one relay node and that the relay node uses the FP scheme for relaying the data from the server to the client. Therefore, Equation (7), for a single-relay SRC using the FP relaying scheme, becomes:

(8) $$G_{t}=\min(G(r,s),G(r,c))$$

Here, *G*(*r*, *s*) and *G*(*r*, *c*) are the goodput of the server-relay and relay-client links, respectively. Figure 3b indicates how the measured goodput changes as the relay node moves between the server and the client nodes in a single-relay SRC framework using the FP scheme.

### 3.3. Problem Formulation

Let us assume that the server and client nodes are mobile (dynamically changes their position) and the relay node has a fully controllable dynamics, but has no control over the server or client nodes' movements. The positions of the server, relay and client nodes are *p_s_*, *p_r_*, and *p_c_*, respectively, in a 2D domain (ℝ^2^), respectively. We also assume an unknown, noisy and distributed radio signal environment (RSS field) and suppose that the RSS can be measured by the relay node using on-board wireless receivers (which act as the RSS sensors). We formulate the problem statement as follows:

**Problem 1**: Find an optimization objective function that should be maximized by optimizing *p_r_*, such that the network capacity (goodput) of the end-to-end server-client link is optimal.
**Problem 2**: Find a robust stochastic optimization (RSO) algorithm that maximizes the objective function and is robust to the dynamics in the environment.
**Problem 3**: Find an RSS sensing method and RSS pre-processing techniques to increase the robustness of the RSO algorithm.
**Problem 4**: Find a suitable dynamics controller for the relay node that supports the solutions of Problems 1–3 and also ensures that the relay node can reach the objective smoothly.

The optimization algorithm is implemented on the relay node, and the inputs to the algorithm are the position (*p_r_*) value of the relay node obtained using an odometer (however, we assume no knowledge on the positions of the server or client nodes) and the RSS values at the relay node for the radio signals received from the server and client nodes. The robustness of our algorithm is verified for objective maximization under sensory and environmental noises. In this regard, simulations and experimental evaluations are conducted considering various environmental conditions.

## 4. Proposed Solution

In this section, we first define an optimization problem based on our objective, and then, we come up with an objective function that satisfies the optimization problem statement. Then, we propose a suitable optimization method and a multi-sensor sampling method (which provides RSS gradient inputs to the optimization algorithm). Then, we present a position controller that implements the optimization algorithm. The algorithm itself is summarized at the end of this section.

### 4.1. Optimization Problem

Let the measured RSS at the relay node from the client node be $RSS_{c}=RSS_{c}^{fs}(\left\|p_{r}-p_{c}\right\|)+\Phi_{c}^{sh}(p_{r},p_{c})+\Phi_{c}^{mp}(p_{r},p_{c},t)$and from the server node be $RSS_{s}=RSS_{s}^{fs}(\left\|p_{r}-p_{s}\right\|)+\Phi_{s}^{sh}(p_{r},p_{s})+\Omega_{s}^{mp}(p_{r},p_{s},t)$. A way to solve Problem 1 is to find a function that maximizes both the *RSS_c_* and *RSS_s_* at the same time, so that the total goodput is at a maximum. Figure 3b shows the RSS readings as the relay node travels between the server and client nodes. The reason for non-symmetrical RSS values between the two nodes is that the environment has NLOS conditions and, hence, has different propagation constants. It can be observed from this figure that the goodput is maximal when *RSS_s_* and *RSS_c_* become close to each other, and this is the basis for the objective of this work, *i.e.*, to maximize the network capacity by maintaining a balance between radio signal strengths received from the server and the client nodes while attempting to maximize the RSS from both sides at the same time. It is also worth noting that this figure shows a one-dimensional RSS measurement, and if extrapolated to a 2D scenario, there can be multiple possible positions where *RSS_c_* and *RSS_s_* are equal, as will be shown later in Section 5. Hence, it is important to consider a function that maximizes the values of *RSS_c_* and *RSS_s_* together while maintaining them as close as possible. The objective of the optimization problem can be formulated as follows:

(9) $$p_{r}^{*}=\underset{p_{r}}{\arg\max}f(p_{r});s.t.G(p_{r}^{*})=\underset{p_{r}}{\max}G(p_{r})\text{and}RSS_{c}(p_{r}^{*})≐RSS_{s}(p_{r}^{*}),\forall p_{r}\in [p_{s},p_{c}]$$

where *G*(*p_r_*) is the goodput value when the relay node is at position *p_r_*. The position of the relay node *p_r_* = (*x_r_* ∈) (ℝ,*y_r_* ∈) ℝ)) (whose value ranges between the positions of server and client nodes) is the control variable (argument), and the function *f*(*p_r_*) : ℝ*^n^* → ℝ is the objective function to be maximized in the optimization problem. $p_{r}^{*}$is the position at which the objective function *f*(*p_r_*) is maximum and also the following two constraints are achieved: maximum goodput; *RSS_c_* and *RSS_s_* values match each other.

### 4.2. Objective Function

A simple function that satisfies the objective of simultaneously maximizing the RSS values from both the server and client nodes would be *f*(*p_r_*) = *RSS_c_*(*p_r_*) + *RSS_s_*(*p_r_*); however, this results in multiple optimum positions, each resulting in a sub-optimum solution. Thus, a different objective function that satisfies all of the constraints has to be chosen. For example, Dixon and Frew [16] used *f*(*p_r_*) = *min*{*SNR_c_*(*p_r_*), *SNR_s_*(*p_r_*)} as the objective function for a similar problem statement and adopted a least squares gradient estimation (LSGE) method for determining the gradient of the objective function and to deal with the non-smooth nature of the objective function.

As our algorithm will rely purely on the spatial gradient approximation (using measurements), determining the gradients for solving a non-smooth objective function has many practical difficulties [35]. To aid the easier integration of our proposed method, we approximate the non-smooth objective function used in [16] to a smooth valued function by using a smoothing approximation method proposed in [36]. Furthermore, as we do not have direct SNR measurements, we use RSS measurements instead. Hence, we propose the following objective function that solves the optimization problem defined in the Equation (9):

(10) $$f(p_{r})=-\log(\exp(-RSS_{c}(p_{r},p_{c}))+\exp(-RSS_{s}(p_{r},p_{s}))\text{where}p_{r}\in \{p_{\text{min}},p_{\text{max}}\}\in {\mathbb{R}}^{2}$$

### 4.3. Optimization Method

The proposed function *f* : ℝ → ℝ^2^ (with bounded RSS values) possesses at least one maximum value (according to the extreme value theorem). In this section, we show how this maximum value can be achieved using an appropriate optimization method. We are inclined towards gradient-based optimization methods for the advantages, such as the convergence and stability, as mentioned in [16]. Optimization methods using only an approximated gradient (of the objective function) information typically use stochastic optimization algorithms [16,37].

The gradients of the objective function are estimated using the RSS measurements and are then used to determine the future position of the relay robot, which maximizes the value of the objective function. A stochastic gradient ascent (SGA) algorithm is used to recursively update the relay node's position based on the RSS gradients as follows:

(11) $$\begin{array}{l} {x_{r}}^{i+1}=x_{r}^{i}+\gamma g_{x}({{\vec{p}}_{r}}^{i})\\ {y_{r}}^{i+1}=y_{r}^{i}+\gamma g_{y}({{\vec{p}}_{r}}^{i}) \end{array}$$

The relay node's position vector ${{\vec{p}}_{r}}^{i}=(x_{r}^{i},y_{r}^{i})$is updated at every *i*−*th* iteration. *γ* ∈ ℝ_+_ is the learning rate (also called “step size”), whose value will be determined in real time in each iteration. The selection of this learning rate is crucial to obtain fast convergence [38] and should satisfy the conditions Σ*_i_γ_i_* = ∞ and $\sum_{i}\gamma_{i}^{2}<\infty$. The choice of the learning rate *γ* will be discussed in Section 4.5.1. The multi-variate gradient vector *g⃗* = (*g_x_*, *g_y_*) of the objective function is determined in the following way:

(12) $$\begin{array}{l} g_{x}=\nabla_{x}f=\frac{\partial f}{\partial x}=\frac{\partial f}{\partial (RSS_{c})}\cdot \frac{\partial (RSS_{c})}{\partial x}+\frac{\partial f}{\partial (RSS_{s})}\cdot \frac{\partial (RSS_{s})}{\partial x}\\ g_{y}=\nabla_{y}f=\frac{\partial f}{\partial y}=\frac{\partial f}{\partial (RSS_{c})}\cdot \frac{\partial (RSS_{c})}{\partial y}+\frac{\partial f}{\partial (RSS_{s})}\cdot \frac{\partial (RSS_{s})}{\partial y} \end{array}$$

(13) $$\text{where},\frac{\partial f}{\partial (RSS_{c})}=\frac{e^{RSS_{s}}}{e^{RSS_{c}}+e^{RSS_{s}}}\text{and}=\frac{\partial f}{\partial (RSS_{s})}=\frac{e^{RSS_{c}}}{e^{RSS_{c}}+e^{RSS_{s}}}$$

The above method is called the stochastic gradient ascent method, because the objective function *f* has both deterministic and stochastic parts, as it is a function of RSS (Section 3.1), and therefore, the stochastic gradient vector is $\vec{g}(p_{r})=\vec{\nabla f}(p_{r})+\vec{\varepsilon }$. The *ε⃗* is the noise vector in the gradient measurements at every iteration. The RSS gradient vector *g⃗* is approximated by measurements at a single position of the relay node. Thus, we try to increase robustness and achieve fast convergence by using the concept of stochastic gradient ascent [39].

The SGA method requires that the objective function *f*(*p_r_*) is concave and the gradient *g*(*p_r_*) is locally Lipschitz continuous to guarantee convergence [37] and, thus, to reach the global optimum value. We show below how the convergence requirements for the SGA method are met.

Proof for concavity: to prove that *f* is concave, it is necessary to show that the Hessian matrix (second-order partial derivatives) is negative semi-definite (NSD) (refer to Remark 2.2.11 in [40]). We have the Hessian matrix,

(14) $$H=\nabla^{2}(f)=\left[\begin{array}{cc} \frac{\partial^{2}f}{\partial x^{2}} & \frac{\partial^{2}f}{\partial x\partial y}\\ \frac{\partial^{2}f}{\partial y\partial x} & \frac{\partial^{2}f}{\partial y^{2}} \end{array}\right]=\left[\begin{array}{cc} -\frac{\exp(RSS_{c}+RSS_{s})}{{\left(\exp(RSS_{c})+\exp(RSS_{s})\right)}^{2}} & \frac{\exp(RSS_{c}+RSS_{s})}{{\left(\exp(RSS_{c})+\exp(RSS_{s})\right)}^{2}}\\ \frac{\exp(RSS_{c}+RSS_{s})}{{\left(\exp(RSS_{c})+\exp(RSS_{s})\right)}^{2}} & -\frac{\exp(RSS_{c}+RSS_{s})}{{\left(\exp(RSS_{c})+\exp(RSS_{s})\right)}^{2}} \end{array}\right]$$

the determinant of the Hessian matrix is zero, and both $\frac{\partial^{2}f}{\partial x^{2}}$and $\frac{\partial^{2}f}{\partial y^{2}}$are negative. This means that the Hessian matrix is NSD (as all the eigenvalues are non-positive for any *RSS_c_* and *RSS_s_* value). Thus, the function *f* is concave.

Proof for Lipschitz continuity: to show that the gradient function ∇*f*(*p_r_*) is locally Lipschitz continuous, it should be proven that there exists a positive constant *L* ≥ 0, such that ‖∇*f*(*RSS^t^*^+1^) − ∇*f*(*RSS^i^*)‖ ≤ *L*‖*RSS^i^*^+1^ − *RSS^i^*‖ in a bounded interval for *RSS* ∈ (*RSS_min_*, *RSS_max_*). Alternatively, one can prove this by showing that the derivative of ∇*f* (which is ∇^2^*f*) exists and that ∇^2^*f* is continuous and bounded in the same interval of the RSS (using the theorem, a bounded derivative implies Lipschitz continuity [41]). We can come to this conclusion by analyzing ∇^2^*f* in Equation (14) and noting that in a bounded interval of the RSS (*RSS_min_*, *RSS_max_*) set by the threshold limits, the function ∇^2^*f* ≤ *K* is bounded with a constant *K* depending on the RSS interval.

Stochastic optimization methods can work with noisy gradient measurements, which is a vital requirement, because the RSS measurements usually contain random variations that can be treated as a measurement noise in the optimization problem. Therefore, the key factor driving the choice of a stochastic optimization method (as a solution to Problem 2) is that the environmental factors affecting the RSS are not known prior, and hence, an optimization method that is both adaptable (to environmental changes) and robust (to noise) is needed.

Though we can reach the global optimum if *f*(*x*) is concave, the stochastic nature of the RSS is not easing this phenomenon, and hence, the objective function has multiple local optima. With careful pre-processing techniques discussed in the following subsection, the local optima issue can be mitigated. Nevertheless, as the objective is to reach the optimum in a localized region, the issue of global optimization is not a concern.

### 4.4. Pre-Processing of the RSS Data

As the measured RSS values are noisy [42], we propose spatial and temporal smoothing RSS filters to mitigate measurement noise and multipath fading effects.

#### 4.4.1. Spatial Smoothing

It has been shown in [43] that the (spatial) multipath fading effects at each sensor can be mitigated by linear spatial averaging of the RSS measurements with several measurements per wavelength (*λ*) of the radio signal used. Moreover, it is mentioned in [44] that there should be a minimum spacing of 0.38*λ* between two RSS spacial samples to obtain independent uncorrelated measurements. Therefore, we propose to use an average filter for RSS spatial smoothing when the relay node is moving from (*x^i^*, *y^i^*) to (*x^i^*^+1^, *y^i^*^+1^). Assuming that we use the 2.4GHz (*λ* = 12.5cm) radio frequency band, the spatial sampling (note: the spatial sampling here refers to the sampling (in space) of the RSS by each sensor on the relay node, whereas the purpose of the spacing of multiple sensors within the relay node is to estimate the RSS gradient around the relay node) frequency (*f_s_*) has been set to a value greater than or equal to 5 cm, meeting both the minimum spacing for uncorrelated measurements, as well as the resolution needs of the linear spatial averaging (around 2.5 measurements per *λ*). The spatial averaging filer is modeled as follows:

(15) $$RSS=\sum_{k=1}^{N}\text{RSS}(x^{k},y^{k}),\text{where}N=\frac{\left\|\left((x^{i+1},y^{i+1})-(x^{i},y^{i}\right)\right\|}{f_{s}}$$

At each receiver, the RSS sampling time is *t_s_* = *f_s_ν*, *ν* being the velocity of the relay node. This means that the outcome of the smoothed RSS will be the RSS sample at $\left(x^{i+\frac{1}{2}},y^{i+\frac{1}{2}}\right)$instead of (*x^i^*^+1^, *y^i^*^+1^). As we are concerned only about the RSS gradient measurements, this spatial averaging has a positive impact [19].

#### 4.4.2. Temporal Smoothing

In a radio receiver, active analog filters, such as an automatic gain control (AGC) circuit, adjust the input amplification gain depending on the received signal level to protect against large signal interference and attenuate slow changes in the received signal strength caused by shadowing effects [45]. As the objective is to mitigate fast multipath fading effects in the RSS, active analog filters are not suitable, and therefore, we propose a digital exponential moving average (EMA) filter for smoothing rapid variations in the RSS over a given time. The filter implementation is similar to a discrete first order infinite impulse response (IIR) or a single-pole low-pass filter that has a recursive feedback. The filter is characterized by the following equation:

(16) $$RSS^{i}=\alpha RSS^{i}+(1-\alpha )RSS^{i-1}=RSS^{i-1}+\alpha (RSS^{i}-RSS^{i-1})$$

where *α* is the smoothness parameter (0 ≤ *α* ≤ 1) that yields the filter time constant $\tau_{f}=\Delta_{T}\left(\frac{1-\alpha }{\alpha }\right)$and Δ*_T_* is the RSS sampling period used in the temporal smoothing.

The next sub-section discusses how to determine the RSS gradients (which is a key ingredient to the SGA method) after applying spatial and temporal filters for RSS smoothing.

### 4.5. Multi-Sensor Sampling

#### 4.5.1. Finite Differences

A fundamental attribute of the stochastic optimization algorithm is the 2D RSS gradient vector $\vec{\nabla \text{RSS}}=(\nabla_{x}\text{RSS},\nabla_{y}\text{RSS})=\left(\frac{\partial \text{RSS}}{\partial x},\frac{\partial \text{RSS}}{\partial y}\right)$, which can be estimated using spatially-distributed RSS measurements from sensors on-board the relay robot. As our intention is to provide a sampling method that is both simple to implement (in different hardware platforms) and reliable to guarantee performance, we make use of multiple wireless transceivers as sensors to enhance reliability and redundancy in wireless networking and implicitly use the concept of multi-sensor spatial diversity to measure RSS gradients instead of methods, such as the least squares gradient estimation (LSGE) method [16,19], search-based methods [46] and multi-antenna spatial diversity techniques [20,23,27].

**Figure 4.**
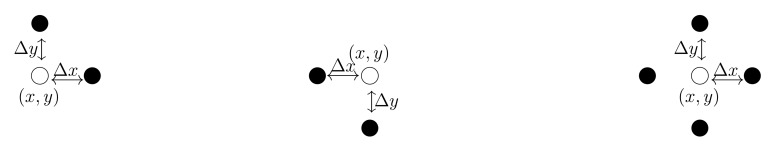
Gradient approximation schemes: backward finite differences (BFD) **(left);** forward finite differences (FFD) **(center)** and central finite differences (CFD) **(right)**.

Finite difference methods, such as forward finite differences (FFD), backward finite differences (BFD) and central finite differences (CFD) are typically used to estimate the gradient using measurements that are spatially distributed about a central point [38]. The RSS gradients are determined using four sensors distributed spatially around the center of the relay node to measure the RSS from the server and client nodes (*RSS_s_* and *RSS_c_*) at appropriate spacing, as shown in Figure 4. The setup also requires a benchmark RSS sample from both the server and client nodes (denoted as $\bar{RSS_{c}}$and $\bar{RSS_{s}}$) at the center of the relay node to determine the direction of change in the RSS. This benchmark value can alternatively be determined using interpolation of the RSS from four distributed sensors, provided they are positioned with geometrical symmetry.

The RSS gradients of the client node [∇*_x_RSS_c_*, ∇*_y_RSS_c_*] and of the server node [∇*_x_RSS_s_*, ∇*_y_RSS_s_*] can be obtained using the derivative approximations with the CFD algorithm, as shown below:

(17) $$\begin{array}{l} \nabla_{x}RSS_{c}=\frac{\partial RSS_{c}}{\partial x}≐\frac{RSS_{c}(x+\Delta x,y)-RSS_{c}(x-\Delta x,y)}{2\Delta x}\\ \nabla_{y}RSS_{c}=\frac{\partial RSS_{c}}{\partial y}≐\frac{RSS_{c}(x,y+\Delta y)-RSS_{c}(x,y-\Delta y)}{2\Delta y}\\ \nabla_{x}RSS_{s}=\frac{\partial RSS_{s}}{\partial x}≐\frac{RSS_{s}(x+\Delta x,y)-RSS_{s}(x-\Delta x,y)}{2\Delta x}\\ \nabla_{y}RSS_{s}=\frac{\partial RSS_{s}}{\partial y}≐\frac{RSS_{s}(x,y+\Delta y)-RSS_{s}(x,y-\Delta y)}{2\Delta y} \end{array}$$

where Δ*x* and Δ*y* are the spacing of the RSS sensors along the x- and y-axis, respectively, on the relay node. The sensors are placed at (*x* + Δ*x*, *y*),(*x* − Δ*x*, *y*),(*x*, *y* + Δ*y*) and (*x*, *y* − Δ*y*) with the benchmark sensor being at (*x*, *y*) on the relay node in the CFD scheme. The authors in [38] show that the CFD method is more accurate than the FFD method, and in the CFD scheme, the stochastic error in gradient estimation is four-times lower than the FFD scheme. We have already experimentally proven this fact in our previous work [15] and have shown that the CFD algorithm had better performance than the FFD algorithm. Hence, we propose to use the CFD algorithm as a solution to Problem 3. Nevertheless, the FFD or the BFD algorithms can still be used for redundancy (see Section 4.5.2).

To minimize errors due to noise in the gradient measurements, the gradient measurements are normalized as follows:

(18) $$\begin{array}{l} \left\|\nabla_{x}RSS_{c}\right\|=\frac{\nabla_{x}RSS_{c}}{\sqrt{{\left(\nabla_{x}RSS_{c}\right)}^{2}+\left(\nabla_{y}RSS_{c}\right)}};\left\|\nabla_{y}RSS_{c}\right\|=\frac{\nabla_{y}RSS_{c}}{\sqrt{{\left(\nabla_{x}RSS_{c}\right)}^{2}+{\left(\nabla_{y}RSS_{c}\right)}^{2}}}\\ \left\|\nabla_{x}RSS_{s}\right\|=\frac{\nabla_{x}RSS_{s}}{\sqrt{{\left(\nabla_{x}RSS_{s}\right)}^{2}+{\left(\nabla_{y}RSS_{s}\right)}^{2}}};\left\|\nabla_{y}RSS_{s}\right\|=\frac{\nabla_{y}RSS_{s}}{\sqrt{{\left(\nabla_{x}RSS_{s}\right)}^{2}+{\left(\nabla_{y}RSS_{s}\right)}^{2}}} \end{array}$$

Applying the CFD, the gradient optimization method shown in the Equation (11) becomes:

(19) $$\begin{array}{l} {x_{r}}^{i+1}=x_{i}^{i}+\gamma_{c}^{i}\frac{\partial f}{\partial (RSS_{c})}\cdot \left\|\nabla_{x}RSS_{c}\right\|+\gamma_{s}^{i}\frac{\partial f}{\partial (RSS_{s})}\cdot \left\|\nabla_{x}RSS_{s}\right\|\\ {y_{r}}^{i+1}=y_{r}^{i}+\gamma_{c}^{i}\frac{\partial f}{\partial (RSS_{c})}\cdot \left\|\nabla_{y}RSS_{c}\right\|+\gamma_{s}^{i}\frac{\partial f}{\partial (RSS_{s})}\cdot \left\|\nabla_{y}RSS_{s}\right\| \end{array}$$

where $\gamma_{c}^{i}$and $\gamma_{s}^{i}$are the learning rates of the gradient algorithm (at the *i*−*th* iteration) from the client and server sides, respectively. The values of the learning rates *γ_c_* and *γ_s_*, are set to be high at initial iterations and low at the end of convergence [46]. A natural choice for the learning rates that exhibit such behavior are $\gamma_{c}=\frac{\kappa }{RSS_{c}^{2}}$and $\gamma_{s}=\frac{\kappa }{RSS_{s}^{2}}$to enable the accelerated convergence when the relay node is farther from the optimum (lower RSS) and decelerated convergence and stabilization when the optimum (higher RSS) has almost been reached. The values of the learning rate constant *κ* is selected empirically. This learning rate selection will make sure the convergence to a local minimum as the learning rate asymptotically satisfies $\overset{\infty }{\underset{j=1}{\text{∑}}}\gamma_{j}\to \infty$and *γ_j_* → 0 as *j* → ∞.

Further, as provided in [19], the RSS gradient can be estimated with least squares given no direct measurements of the spatial gradient.

(20) Xβ=Z

(21) $$\beta ={\left(X^{T}X\right)}^{-1}X^{T}Z$$

For *X^T^X* to be non-singular for a unique solution of *β*, geometrically, the spatial samples must be co-linear, which is inherent in the CFD sampling.

#### 4.5.2. Redundancy

While it might at first sight look as if there is no inherent redundancy in this scheme, there are multiple ways to deal with failing devices, and the fact that five wireless devices are present can be exploited as a redundancy feature. For this, it is necessary to acknowledge that the gradient measures in formulas (17) are approximated using CFD, but can also be approximated using FFD or BFD.

As an example, we treat the case where the wireless device at location (*x* + Δ*x*) is broken for measurements. This will have an impact on the approximation of ∇*_x_RSS_c_* and ∇*_x_RSS_s_*. However, using BFD, Formulas (17) and (19) can be replaced by:

(22) $$\nabla_{x}RSS_{c}≐\frac{RSS_{c}(x,y)-RSS_{c}(x-\Delta x,y)}{\Delta x}$$

and:

(23) $$\nabla_{x}RSS_{s}≐\frac{RSS_{s}(x,y)-RSS_{s}(x-\Delta x,y)}{\Delta x}$$

respectively. A similar substitution can be performed for the case where other devices fail. It can easily be seen that any one of the devices can fail and the functionality recovered using another gradient approximation scheme.

### 4.6. Controller Design

**Figure 5.**
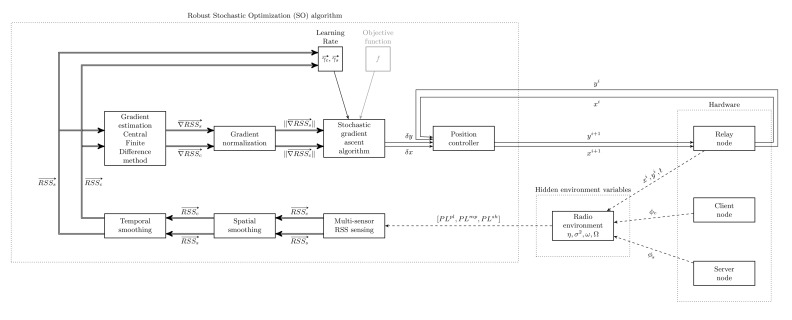
Schematic view of the proposed position controller implementing the robust stochastic optimization algorithm.

The output of the optimization method is a new position vector ${\vec{p_{r}}}^{i+1}=({x_{r}}^{i+1},{y_{r}}^{i+1})$for the relay robot. A controller should be designed to move the relay robot to the new position. A closed-loop incremental position controller, as shown in Figure 5, is devised as the solution for Problem 4. The RSO algorithm consists of the following sub-modules: multi-sensor RSS sensing, temporal and spatial smoothing, gradient estimation using the CFD method, gradient normalization and the SGA algorithm. The motion dynamics is controlled using a position controller acting on the relay node. The radio environment variables are unknown (hidden) to the algorithm, as indicated in Figure 5. The controller is stopped on meeting both of the following two conditions:

- the difference in the benchmark values of the RSS from the server and client nodes is less than a threshold value, $\left|\bar{RSS_{c}}-\bar{RSS_{s}}\right|<{\text{RSS}}_{\text{difference}}$;
- the *l*^2^ norm of the gradient is less than a gradient threshold (which is close to zero), ‖*g*‖ < *g*_difference_.

In addition to the algorithmic parameters, such as Δ*x*, Δ*y*, *κ*, and stop criterion parameters, such as RSS_difference_, *g*_threshold_, the inputs to the algorithm are relay node position vector *p⃗_r_* and five RSS measurements each for server and client sides. The next sub-section presents the implemented algorithm.

### 4.7. Algorithm

Assuming that the relay node moves on a 2D surface (*X*, *Y*) with the current position of the relay node as (*x^i^*, *y^i^*), the RSS values at five receivers on the relay node from the server node as *RSS_s_*(*x^i^* ± Δ*x*, *y^i^* ± Δ*y*) and from the client node as *RSS_c_*(*x^i^* ± Δ*x*, *y^i^* ± Δ*y*) at instant *i*, the robust stochastic optimization algorithm is expressed in Algorithm 1. The aim of the algorithm is not to reach the global optimum in one iteration, but to reach the global optimum (within a local region) progressively after several iterations. The proposed algorithm measures only the relative direction of the nodes and uses RSS measurements only in a very basic and, thus, robust way.

Sensors spacing intervals (Δ*x* and Δ*y*) depend on the physical constraints of the relay node. For instance, in the youBot mobile robot that is used in the experiments, the feasible maximum values are Δ*x* ≤ 0.38 m and Δ*y* ≤ 0.58 m. *RSS*_threshold_ is an important factor that determines when to start the algorithm, because only when the current RSS is below this threshold, improving the RSS will be fruitful, as can be inferred from Figure 3. On the other hand, the algorithm stops when the difference between the RSS values from the server and client nodes reaches a threshold value *RSS*_difference_ and the gradient norm is less than a threshold value *g*_threshold_.

It is important to note that we use the RSS gradient information (instead of SNR gradients used in [16]) and propose a suitable robust stochastic optimization algorithm to optimize the relay node position for receiving a relatively better RSS (relative maxima), thereby increasing the communication range and the network capacity.

**Algorithm 1** Robust stochastic optimization algorithm for RSS balancing between two wireless nodes.
1:Inputs: *x^i^*, *y^i^*, Δ*x*, Δ*y*, *κ*, *RSS*_threshold_, *RSS*_difference_, *g*_threshold_2:Outputs: *x^l^*^+1^, *y^l^*^+1^3:**if** (
$\bar{RSS_{s}}<RSS_{\text{threshold}}$or 
$\bar{RSS_{c}}<RSS_{\text{threshold}}$) **then**4: **while**
$\left(\left|\bar{RSS_{s}}<\bar{RSS_{c}}\right|≰RSS_{\text{difference}}\right)$and (‖*g*‖ ≰ *g*_threshold_) **do**5:  Multi-sensor spatial sampling and filtering ()6:
$\vec{RSS_{c}}=[RSS_{c}(x^{i}\pm \Delta x),RSS_{c}(y^{i}+\Delta y),\vec{RSS_{c}}(x^{i},y^{i})]$7:
$\vec{RSS_{s}}=[RSS_{s}(x^{i}\pm \Delta x),RSS_{s}(y^{i}\pm \Delta y),\vec{RSS_{s}}(x^{i},y^{i})]$8:
$\left[\vec{RSS_{c}},\vec{RSS_{s}}]=\text{Spatial Filtering}(\vec{RSS_{c}},\vec{RSS_{s}}\right)$9:
$\left[\vec{RSS_{c}},\vec{RSS_{s}}]=\text{Temporal Filtering}(\vec{RSS_{c}},\vec{RSS_{s}}\right)$10:  RSS Gradient Estimation ()11:
$\nabla_{x}RSS_{c}=\frac{RSS_{c}(x+\Delta x)-RSS_{c}(x-\Delta x)}{2\Delta x},\nabla_{y}RSS_{c}=\frac{RSS_{c}(y+\Delta y)-RSS_{c}(y-\Delta y)}{2\Delta y}$12:
$\nabla_{x}RSS_{s}=\frac{RSS_{s}(x+\Delta x)-\bar{\bar{RSS_{s}}}(x-\Delta x)}{2\Delta x},\nabla_{y}RSS_{s}=\frac{\bar{\bar{RSS_{s}}}(y+\Delta y)-\bar{\bar{RSS_{s}}}(y-\Delta y)}{2\Delta y}$13:  Gradient Normalization ()14:
$\nabla RSS_{c}=\sqrt{\nabla RSS_{c}^{2}+\nabla_{x}RSS_{c}^{2}},\nabla RSS_{s}=\sqrt{\nabla_{x}RSS_{s}^{2}+\nabla_{x}RSS_{s}^{2}}$15:
$\left\|\nabla_{x}RSS_{c}\right\|=\frac{\nabla_{x}RSS_{c}}{\nabla RSS_{c}},\left\|\nabla_{y}RSS_{c}\right\|=\frac{\nabla_{y}RSS_{c}}{\nabla RSS_{c}}$16:
$\left\|\nabla_{x}RSS_{s}\right\|=\frac{\nabla_{x}RSS_{s}}{\nabla RSS_{s}},\left\|\nabla_{y}RSS_{s}\right\|=\frac{\nabla_{y}RSS_{s}}{\nabla RSS_{s}}$17:  Stochastic Gradient Descent Algorithm ()18:
$\gamma_{c}=\frac{\kappa }{RSS_{c}^{2}};\gamma_{s}=\frac{\kappa }{RSS_{s}^{2}};\frac{\partial f}{\partial (RSS_{c})}=\frac{e^{RSS_{s}}}{e^{RSS_{c}}+e^{RSS_{s}}};\frac{\partial f}{\partial (RSS_{s})}=\frac{e^{RSS_{c}}}{e^{RSS_{c}}+e^{RSS_{s}}};$19:
$g_{x}(\text{client})=\frac{\partial f}{\partial (RSS_{c})}\cdot \nabla_{x}RSS_{c};g_{x}(\text{server})=\frac{\partial f}{\partial (RSS_{s})}\cdot \nabla_{x}RSS_{s}$20:
$g_{y}(\text{client})=\frac{\partial f}{\partial (RSS_{c})}\cdot \nabla_{y}RSS_{c};g_{y}(\text{server})=\frac{\partial f}{\partial (RSS_{s})}\cdot \nabla_{y}RSS_{s}$21:*g_x_= g_x_*(*client*) + *g_x_*(*server*); *g_y_* = *g_y_*(*client*) + *g_y_*(*server*)22:
$\vec{g}=(g_{x},g_{y});\left\|g\right\|=\sqrt{g_{x}^{2}+g_{y}^{2}}$23:*δx* = *γ_c_g_x_*(*client*) + *γ_s_g_x_*(*server*); *δy* = *γ_c_g_y_*(*client*) + *γ_s_g_y_*(*server*)24:  Relay Node Position Controller ()25:   *x^i^*^+1^ = *x^i^* + *δx*26:   *y^i^*^+1^ = *y^i^* + *δy*27:  Move the relay node to position (*x^i^*^+1^, *y^i^*^+1^)28: **end while**29:**end if**

#### 5. Simulations

The proposed robust stochastic optimization (RSO) algorithm is first evaluated with simulations before moving to field experiments. In this section, we begin with presenting the types of simulation cases, evaluation methods and metrics. Then, we continue to present the simulation setup and finally present the results with analysis. The following simulation cases are considered:

**Case 1: Considering an LOS scenario without spatio-temporal noise**

In this case, we assume that the radio signal is not blocked by large obstacles, and hence, the RSS has a line-of-sight from the source to the destination device. Furthermore, we assume that the RSS spatial variations due to shadowing effects are negligible, as the environment is free of obstacles, and the multipath fading effects on RSS are filtered out using the temporal and spatial filters.

**Case 2: Considering an LOS scenario with spatio-temporal noise**

In Case 2, we use LOS assumptions as in Case 1. However, the RSS spatial variations due to shadowing and multipath effects are included (considered) to some extent, even though we assume that we filter out most of the variations in the RSS (including the measurement noise of the device).

**Case 3: Considering an NLOS scenario with spatio-temporal noise**

In this case, we assume that the radio signals have no direct line-of-sight between wireless devices and suffer blockage by several obstacles in the environment and, hence, qualify for NLOS conditions. Verifying the NLOS conditions is relevant to the algorithm, because the relay node aims to overcome the NLOS barriers in radio propagation and ideally convert the relayed channel from an NLOS link to two LOS links passing through the relay node. The variations in RSS due to shadowing and multipath effects are considered similar to Case 2.

### 5.1. Simulation Setup

A realistic radio signal strength environment is simulated in MATLAB. The pseudo-code in Algorithm 2 shows the principle of the simulation setup. The simulation setup uses the extended log-normal shadowing model given in the Equation (2). There are two transmitter nodes, namely the server and client nodes fixed at positions (−30,0) and (30,0), respectively, in a 100 ×100m 2D space. The receiver is the (mobile) relay node, and its initial position is chosen randomly based on the simulation cases. The distance from the source to the receiver (relay robot) is calculated based on the Euclidean norms.

The radio environment parameters, such as the propagation constant *η* and the path loss values at reference distance *PL_d_*_0_ (which can be quite different for the “server to relay” link and the “client to relay” link), are set based on empirical observations made in [6].

The channel noise in the radio signals are not accurately measurable by most of the commercial wireless transceivers; therefore, we restrict our measurements to only the RSS values (instead of SNR values), although we do present an example simulation scenario where we assume measuring SNR values and consider a localized noise source as in [16,17] in order to show the flexibility of our algorithm.

**Figure 6.**
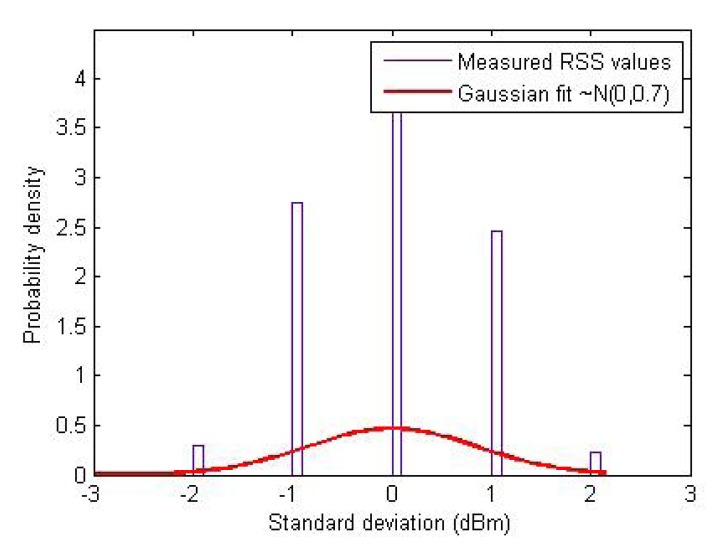
Histogram of RSS variations.

**Figure 7.**
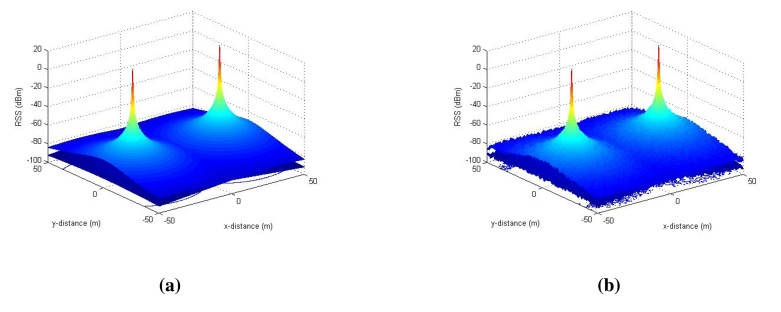
Simulation of the radio environment in 2D. The server node is placed at (30,0), and the client node is placed at (−30,0). (**a**) Simulations without Gaussian noise; (**b**) Simulations with Gaussian noise 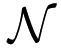
(0, 2).

Figure 6 plots the histogram of the standard deviation of the RSS values measured in a tunnel environment at CERN. As it is clear from this figure that the stochastic parts (shadowing and multipath effects) of the RSS are indeed Gaussian [18], we model them as spatio-temporal noise in the RSS measurements using zero-mean Gaussian random variables (*std_server_* and *std_client_*) in the simulations. Based on the measurements, we found that the standard deviation of the RSS owing to shadowing effects can range from 0.5 dBm to 2 dBm with an average of about 1 dBm at each point in space. Hence, to replicate as realistic an environment as possible in our simulations, we consider the stochastic noise in RSS with up to 4 dBm^2^ variance. A simulated RSS environment for the server and the client nodes is shown in Figure 7 without and with a Gaussian noise (of 4 dBm^2^ variance) in the RSS.

**Algorithm 2** Simulation setup for the RSS environment.
1[x, y] = meshgrid(−50:0.1:50) % Generating a mesh grid of 10^4^m^2^ with 0.1m resolution%(*x_c_*,*y_c_*) and (*x_s_*,*y_s_*) are the positions of the client and server nodes respectively.3*Pt_s_=* 17, *Pt_c_=* 17 % Transmit power (dBm) of the server and client nodes*PL_d_*_0_
*=* 40 % Path loss (dB) at the reference distance (*d*_0_ = 1*m*)5*η_c_* = 2.52, *η_s_* = 3.02 % Path–loss exponent for relay–client and server–relay links*std_server_* = 0 + σ_s_*randn(50, 50, 1) % Spatio–temporal Gaussian noise in server–relay link7*std_client_=* 0 + σ_c_*randn (50, 50, 1) % Spatio–temporal Gaussian noise in relay–client linkfor x in −50 to 509 for y in −50 to 50 if (x == *x_c_* && *y* == *y_c_*) % for relay–client link11  *RSS_c_*(*x*,*y*) = *Pt_c_* else13
$RSS_{c}(x,y)=Pt_{c}-(PL_{d0}-10\eta_{c}\log_{10}(\frac{\sqrt{{\left(x-x_{c}\right)}^{2}+{\left(y-y_{c}\right)}^{2}}}{d_{0}})-std_{\text{client}}(x,y))$end15if (x == *x_s_* && y == *y_s_*) % for server–relay link *RSS_s_*(*x*,*y*) = *Pt_s_*17else
$RSS_{s}(x,y)=Pt_{s}-(PL_{d0}-10\eta_{s}\log_{10}(\frac{\sqrt{{\left(x-x_{c}\right)}^{2}+{\left(y-y_{c}\right)}^{2}}}{d_{0}})-std_{\text{server}}(x,y))$19 end end21end

### 5.2. Simulation Results

Algorithm 1 is made to work in the simulated RSS environment (Algorithm 2), and the performance is evaluated using the following parameters:

**Theoretical optimum**: The position (*x_O_*, *y_O_*) at which the RSS values of both the server and the client nodes are maximum, as well as equal (max *RSS_c_* = max *RSS_s_*) without including any multipath effects (*PL^mp^* = 0). In simulations, the objective function *f* at each point in space can be calculated (because the RSS environment is fully known), and hence, the theoretical optimum is the global optimum (where *f* is maximum), as well. On the other hand, in field experiments, the theoretical optimum is calculated using Equation (1) (but without *χ_σ_*) by substituting the empirical values of *PLd*_0_, *η_c_* and *η_s_* observed in that environment.
**Threshold zone** (m^2^): A circular area with the center as the theoretical optimum *(x_O_*, *y_O_*) and the radius (*r_t_*) equal to 10% of the total distance between the initial relay node position (when the algorithm started) and the theoretical optimum position $\left(r_{t}=0.1\sqrt{{\left(x_{r}^{0}-x_{O}\right)}^{2}+{\left(y_{r}^{0}-y_{O}\right)}^{2}}\right)$, where $\left(x_{r}^{0},y_{r}^{0}\right)$is the initial relay node position).
**MAE (m):** The mean absolute distance error in reaching the theoretical optimum position.
**RMSE (m)**: The root mean squared error in reaching the theoretical optimum position.
**Success rate (%)**: The percentage of success in reaching the threshold zone at the end of the algorithm. When the relay is at its final position $\left(x_{r}^{f},y_{r}^{f}\right)$, success = 100 if $x_{r}^{f^{2}}+y_{r}^{f^{2}}\leq r_{t}^{2}$, and success = 0, otherwise. The success rate is the average value of the success scores over all of the simulation trials in each case.
  **Distance cost (m)**: The total (Euclidean) distance traveled from the initial position to the theoretical optimum position.
**Time cost (#iter)**: Time taken in reaching the optimum, measured indirectly using the number of iterations.
**Speed of convergence** $\left(\frac{m}{\#\text{iter}}\right)$: The speed at which the algorithm reaches the optimum position, measured as $\frac{\text{Distance cost}}{\text{Time cost}}$.

One hundred trial runs are executed in each simulation case. The mean and standard deviation values of the above-mentioned parameters are considered in the performance analysis. The results of each simulation case are presented below.

#### 5.2.1. Case 1: LOS Scenario without Noise

Simulation Case 1 is executed by placing the relay node initially at random locations all around the simulation grid area in each trial. The intention of Case 1 is to understand the distance range of the relay node from the global optimum position over which the proposed algorithm can prove effective. The settings for the parameters used in this case are as follows: propagation constant for relay-client link *η_c_* = 2.52; propagation constant for server-relay link *η_s_* = 2.52; reference path loss at 1-m distance *PL_d_*_0_ = 40 dB; learning rate constant *κ* = 0.01; IIR filter coefficient *α* = 0.8; sensors' separation distance Δ*x* = Δ*y* = 0.2 m; RSS threshold *RSS*_threshold_ = −55 dBm; threshold in difference between the RSS from the server and client nodes *RSS*_difference_ = 2 dBm; gradient threshold *g*threshold = 0.1.

**Figure 8.**
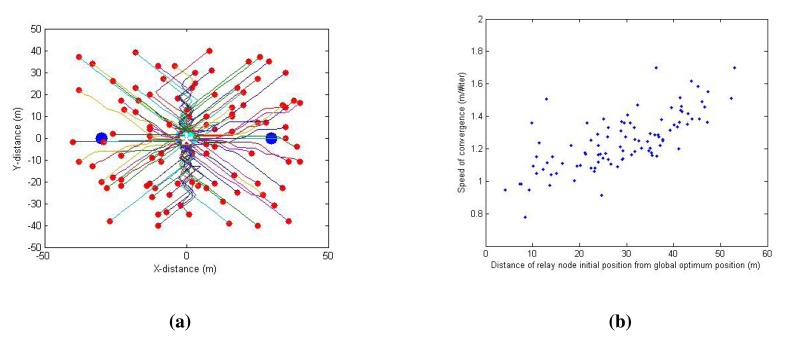
Case 1 simulation results for 100 trials with random relay node initial positions. (**a**) Relay node path from the initial position (red) to the optimum position (green). Blue dots indicate the server and client node locations. Theoretical optimum (*) is at the center (0,0); (**b**) Scatter plot of the distance of initial relay node position from the optimum against the speed of convergence.

Figure 8a depicts the path taken by the relay node from its initial random position (red dots) to the optimum position (green dots) (the client node and server node are shown as blue dots). It can be observed from Figure 8b that the farther the relay node is from the theoretical optimum, the faster the algorithm works, because of the learning rates selection to obtain the optimum convergence rate, as explained in the Section 4.5.1. For Case 1, the MAE value is 1.7 m (the initial relay node is randomly located within a range of 10 to 50 m from the theoretical optimum) and the success rate achieved is 83%.

In reality, the relay node is always at a lower distance range (usually within 15 m) to the global optimum at any given point in time, because the solution using the relay node is implicitly dynamic in nature, and the relay node keeps optimizing its position to reflect the changes in the client or server node positions. Therefore, in further simulations, we fix the relay node initial position at (−15,15) to simulate the realistic conditions.

#### 5.2.2. Case 2: LOS Scenarios with Gaussian Noises

**Figure 9.**
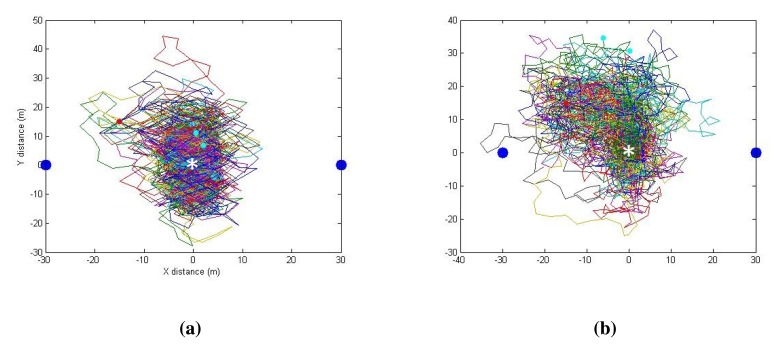
Case 2 simulation results showing 100 trials of two different noise conditions. The theoretical optimum (*) is at the center (0,0). (**a**) LOS scenario with 1 dBm spatio-temporal Gaussian noise in RSS; (**b**) LOS scenario with 1 dBm spatio-temporal Gaussian noise in RSS.

Case 2 is simulated using the same parameters as in Case 1, but with an added Gaussian noise. Two different scenarios are considered, one is using the standard deviation *σ* of the spatio-temporal Gaussian noise as 1 dBm and another uses 2 dBm. The results of the simulations are shown in Figure 9. In both of the plots of Figure 9, the theoretical optimum position without noise is (*x_O_*, *V_O_*) = (0, 0) (because in LOS, the *η_c_* and *η_s_* are same, and therefore, (0,0) is the least equidistant position from both the server and the client nodes). It can be observed that the relay node tries to reach that optimum position. Even though the success rate has dropped compared to Case 1 results due to the addition of noise, we still achieved more than a 75% success rate for the Case 2.

#### 5.2.3. Case 3: NLOS Scenarios with and without Gaussian Noises

For Case 3, we executed six different types of NLOS scenarios with different noise and NLOS conditions. We observed that a difference of 0.5 in *η* in the propagation constants can differentiate between an LOS and an NLOS condition, and a difference of two can differentiate a deep-NLOS condition from an LOS condition [6]. Therefore, in this simulation case, we use different different propagation constants for the relay-client and server-relay link to simulate NLOS conditions. The simulation parameters are similar to Case 2 (*η_c_* = 2.52), but *η_s_* = 3.02 for an NLOS scenario and *η_s_* = 4.52 for a deep-NLOS scenario.

**Figure 10.**
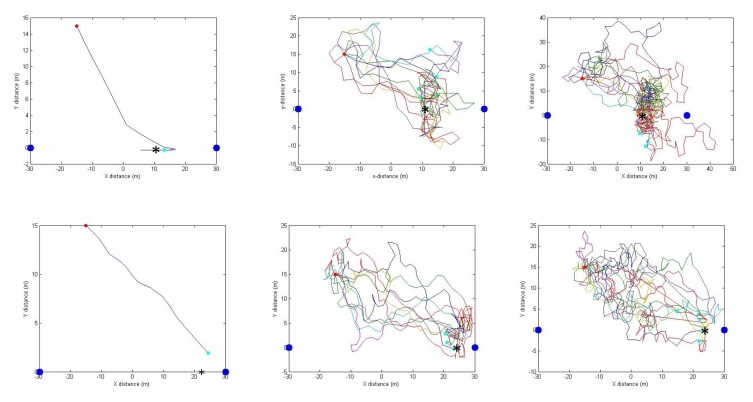
Case 3 simulation results showing 10 trials each. Without noise **(left),** with 1 dBm noise **(center)** and with 2 dBm noise **(right)**. The results of NLOS conditions are presented in the first row, and in the second row, the results of deep-NLOS conditions are presented. The theoretical optimum position is indicated as *.

The results of all six scenarios are presented in Figure 10. The left figures present the NLOS scenarios without noise, so that the optimum position can be observed clearly (which are at (10,0) and (22,0) for NLOS and deep-NLOS scenarios, respectively). As *η_c_* and *η_s_* values are different, the theoretical optimum is not equidistant from the server and the client nodes. The center figure presents the NLOS scenarios with 1 dBm noise. The right figure presents the NLOS scenarios with 2 dBm noise. We present only the first 10 trials of the actual simulation, so that the algorithm behavior can be observed without ambiguity.

**Figure 11.**
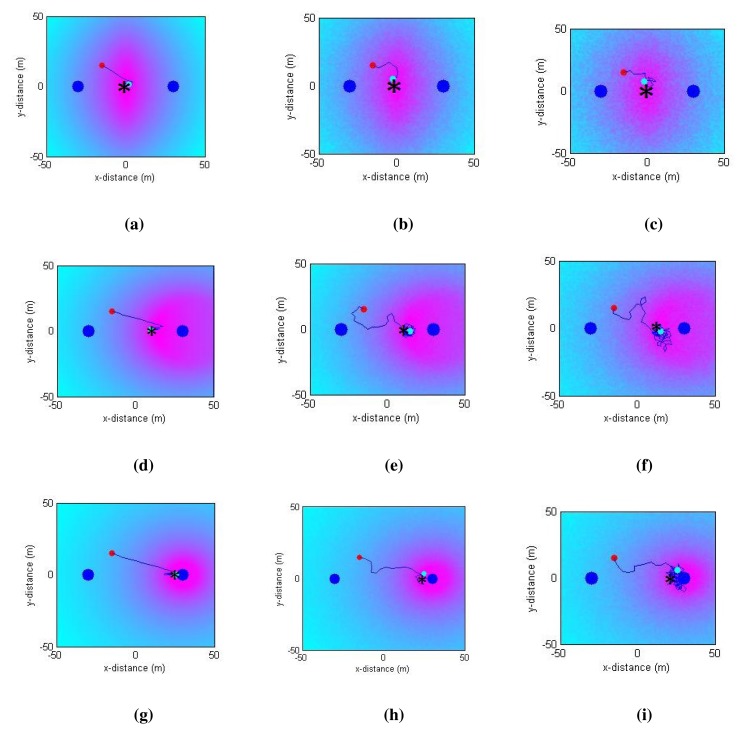
Path taken by the relay node and the objective function value for all scenarios. The theoretical optimum positions are indicated as *. (**a**) LOS, without noise; (**b**) LOS, with 1-dBm noise; (**c**) LOS, with 2-dBm noise; (**d**) NLOS, without noise; (**e**) NLOS, with a 1-dBm noise; (**f**) NLOS, with 2-dBm noise; (**g**) Deep-NLOS, without noise; (**h**) Deep-NLOS, with 1-dBm noise; (**i**) Deep-NLOS, with 2-dBm noise.

The results of the performance metrics for all of the simulation cases are presented in Table 1. One can infer that as the complexity increases, the success rate drops (and the error increases). Figure 11 presents the relay node path for a random run in each simulation case overlaid on the value of the objective function (Equation (10)). The results show promising convergence to the maximum (shown as *) of the objective function value.

**Figure 12.**
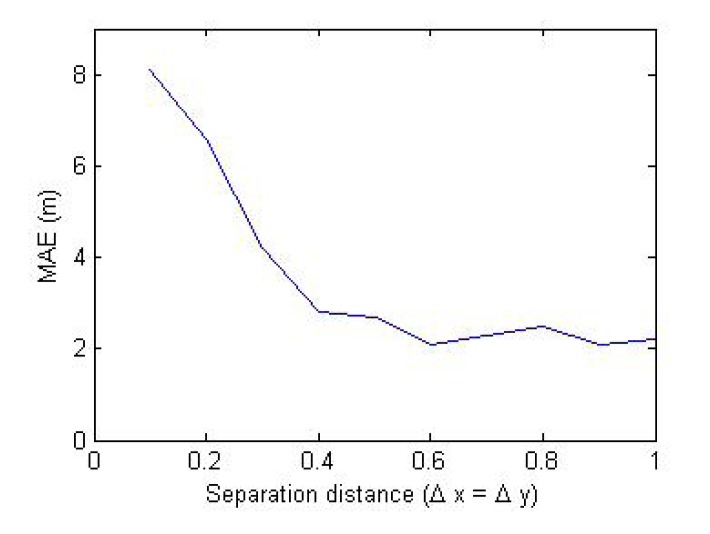
Influence of sensor separation distance on the mean absolute error (MAE).

In Figure 12, the influence of sensor separation on the error for LOS condition with 2 dBm noise are presented, which shows that the higher the sensors' separation, the lower the error in reaching the optimum position. However, the physical size of wireless relay nodes restricts the feasible sensor separation distances.

#### 5.2.4. Considering SNR and a Localized Noise Source

Even though the proposed algorithm is designed for the RSS measurements, we show shortly that it can also handle the SNR measurements without any modifications if the wireless transceivers are capable of measuring noise power (as in [16,17]).

We simulate the RSS as before and simulate a localized external noise source (with the noise power *PN*) in the environment and then derive the SNR using the Equation (3). There can be many external noise sources existing in a radio environment. We provide an example simulation using one external noise source, but the principle can be extended to multiple localized external noise sources.

**Figure 13.**
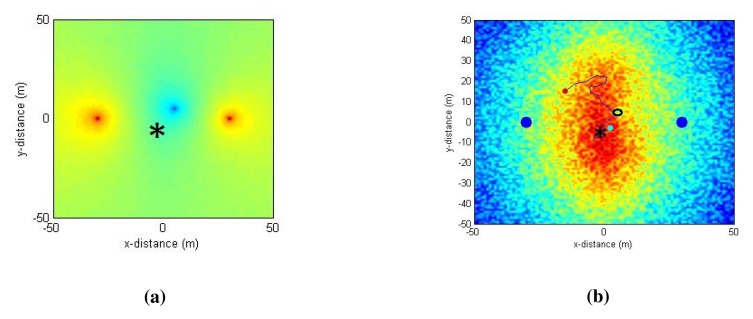
A sample simulation using SNR measurements for the LOS scenario with 2-dBm Gaussian RSS noise and a localized external noise source. The theoretical optimum is indicated as *. (**a**) Map of SNR values. Server node at (−30,0), client node at (30,0) and a localized noise source at (5,5); (**b**) Path taken by the relay node in reaching the global optimum overlaid on the objective function value.

In addition to the simulated settings used before, a localized external noise source (with 10% of the power of the server/client signal) is added at (5, 5) in the environment, as can be seen in Figure 13a. A sample output of simulation Case 2 (LOS and a Gaussian spatio-temporal noise of 2dBm) using the SNR measurements is presented in Figure 13b. The Figure 13 shows the efficiency of the algorithm in avoiding the external noise location and driving the relay node towards the global optimum position adapted to the noisy environment.

## 6. Field Experiments

### 6.1. Experimental Setup

**Figure 14.**
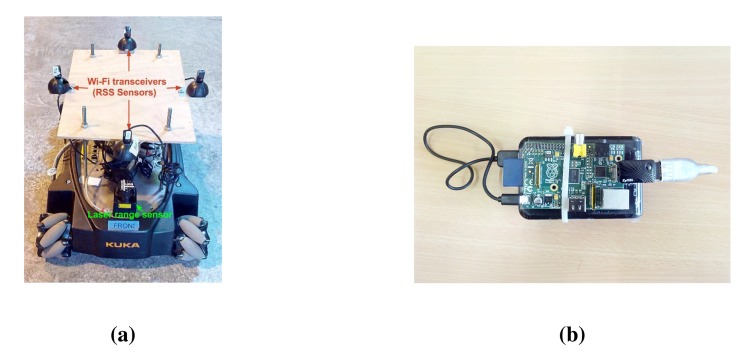
Raspberry Pi and youBot being used as client/server and relay nodes, respectively. (**a**) Multiple spatially-distributed RSS sensors on the relay node; (**b**) Raspberry Pi computer (connected to a battery) used as client or server nodes.

**Figure 15.**
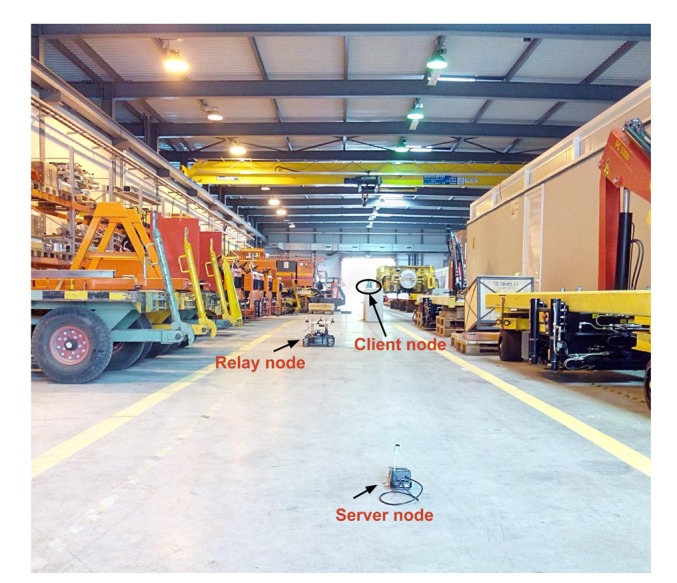
Experimental setup of the server-relay-client nodes at a CERN facility.

Moving from simulations to field experiments, we present the experiments carried out in CERN's experimental test facilities. A youBot [47] mobile robot platform (shown in Figure 14a) is used as the relay node, and two stand-alone Raspberry Pi mini-computers (shown in Figure 14b) are used as the server and client nodes. As the youBot has omni-directional wheels, navigation is efficient given the space restrictions in constrained spaces (hostile environments). Figure 15 shows the experimental setup in an indoor facility containing large metallic objects. Due to the nature of the experiments and the confined space in the environment, the need to have an obstacle avoidance feature for the relay node is inevitable. We detect obstacles within a 4-m range of the robot platform using a 2D laser range (LiDAR) sensor and make use of a basic obstacle avoidance system using the vector field histogram technique [48].

As the robot platform used is velocity-controlled, the position controller part of the algorithm is modified to a velocity controller using state-space transitions. The control equations for the relay node located in the inertial frame of reference with pose (*x*, *y*, *θ*) are given as follows:

(24) $$\left[\begin{array}{c} x_{t}\\ y_{t}\\ \theta_{t} \end{array}\right]=\left[\begin{array}{c} x_{t-1}\\ y_{t-1}\\ \theta_{t-1} \end{array}\right]+\left[\begin{array}{ccc} \pm \vartheta_{X} & 0 & 0\\ 0 & \pm \vartheta_{Y} & 0\\ 0 & 0 & \pm \omega \end{array}\right]\;\left[\begin{array}{c} \tau_{\vartheta }\\ \tau_{\vartheta }\\ \tau_{\omega } \end{array}\right]$$

where *ϑ_x_*, *ϑ_y_* and *ω* are the longitudinal, transverse and rotational velocities, respectively *τ_ϑ_* and *τ_ω_* are time constants that determine the displacements *δx*, *δy* and *δθ* in *x*, *y*, and *θ* directions.

$$\tau_{\vartheta }=\frac{\sqrt{{\left(\delta x\right)}^{2}+{\left(\delta y\right)}^{2}}}{\vartheta }\text{and}\tau_{\omega }=\frac{\delta \theta }{\omega }$$

Because the laser scanner is fitted on the front side of the relay node, the relay robot is made to move only in the forward direction, and therefore, it will first turn towards the necessary control direction and then move to the corresponding position in each iteration. This means that the control output of algorithm has been transferred from the Cartesian space (*x*, *y*) to the polar domain (*r*, *θ*), as follows.

(25) $$\begin{array}{lll} r=\sqrt{x^{2}+y^{2}}; & \theta =\tan^{-1}(y/x); & \text{if}(x<0)\text{then}\theta =\theta +\pi \end{array}$$

The wireless devices used are the following: Edimax EW-7811un WiFi USB card as the “access points (transmitter)” on the client and server nodes and Zyxel NWD2105 WiFi USB card as the “receiver stations” (RSS sensors) on the relay node. The spatial distribution of the RSS sensors on the relay node can be seen in Figure 14a.

Each receiver is set to Channel 3 in the IEEE 802.11n standard 2.4-GHz spectrum, and all of the receivers have the same characteristics (internal omni-directional antenna, receiver sensitivity (*R_X_*) = −182 dBm). They are placed on the relay node with the same orientations to minimize antenna orientation effects in the RSS. The RSSI metric, which directly provides the RSS values in dBm (as the Zyxel NWD2105 uses the RALink 3070 driver), is assessed using the iwconfig command in Linux. Each RSSI sample is measured at a 100-Hz sampling rate, and then, the corresponding temporal and spatial RSS filters are employed, as described in Section 4.4.

The wireless receivers on the relay node are separated with Δ*x* = 16 cm and Δ*y* = 20 cm. This is done to mitigate the effects of interference between various receivers. It also means that the effects of channel noise and inter-receiver interferences are negligible between each RSS spacial sample, as each sample is measured at more than $\frac{\lambda }{2}$spacing. Moreover, we pre-select the best channel for each receiver based on the traffic conditions to avoid adjacent and co-channel interference, and hence, channel optimization is done beforehand.

The settings for the algorithmic parameters used in the experiments were as follows: learning rate constant *κ* = 0.1; IIR filter coefficient *α* = 0.9; sensors' separation distance Δ*x* = 0.16 m, Δ*y* = 0.2 m; *RSS*_threshold_ is set to −130 dBm because of the space restrictions; threshold in difference between the RSS from the server and client nodes *RSS*_difference_ = 2 dBm; gradient threshold *g*_threshold_ = 0.5. Certain constraints in the experimental setup are listed below.

- Due to the fundamental nature of radio signal propagation in physical environments, the theoretical global optimum position where the objective function value is maximum cannot be determined without a dense RSS sampling of the complete environment.
- Because of large metal objects in the environment, the radio reflections from such objects constitute a major part of the intensity in the received signal [6], and hence, the direction of RSS determined using RSS gradients could point to the metal objects themselves instead of pointing to the actual radio source.

The above-mentioned physical constraints and fundamental limitations complicate determining the performance metrics used in Section 5.2, and therefore, we report mainly the success rate (reaching the 10% threshold of theoretical global optimum position) and prove the performance of our algorithm by showing the continuous improvements in the measured RSS values on both client and server sides.

### 6.2. Experimental Results

Over 50 different field trials were conducted in both LOS and NLOS conditions to verify the efficiency of the proposed algorithm. The results of various trials are summarized in Table 2. We obtained an average of a 78% success rate (determined based on the resulting final position and the theoretical optimum position). An average of a 3.5-dBm improvement in RSS is obtained for a displacement of 1 m by the relay node. In the field experiments, the temporal performance is grounded based on the time taken by the algorithm instead of the number of iterations as used in the simulation trials.

The obstacle avoidance system also had some impact on the performance of the algorithm, as it constrained the free control on the motion executed by the algorithm and, in some cases, redirected the robot in a completely opposite direction (inhibiting the directions provided by the algorithm). Therefore, only the results from the trials where the robot was not obstructed or blocked by obstacles are used in the performance analysis. A possible solution to overcome this issue is to use a sophisticated localization system, such as a simultaneous localization and mapping (SLAM) algorithm that provides the layout of the physical objects in the environment, using which the influence on the desired direction of RSO algorithm can be determined, and corrective actions can be taken. However, this is out of scope of this paper and is indeed a possible avenue for further research.

It is important to note that we consider the theoretical optimum as the reference value (for calculating the success rate) in the field experiments, which need not be the actual global optimum due to the physical constraints mentioned earlier. Hence, the percentage of cases that have a zero success score does not necessarily imply that the algorithm failed; however, it could imply that the actual global optimum could be at the position where the relay node finally reached, for instance, because of NLOS influence.

**Figure 16.**
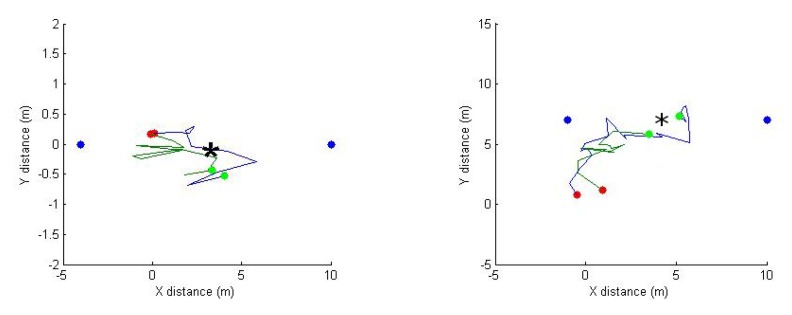
Path taken by the relay node in two different trials when the initial positions are almost the same. Server and client node are shown as blue dots. Red and green dots indicate initial and final relay node position, respectively. The theoretical optimum position is marked as *.

Figure 16 presents two instances of paths taken by the relay node for two similar trials experimented at different times. This is to show that a given radio environment (field) does not change significantly with time because the path taken is similar.

**Figure 17.**
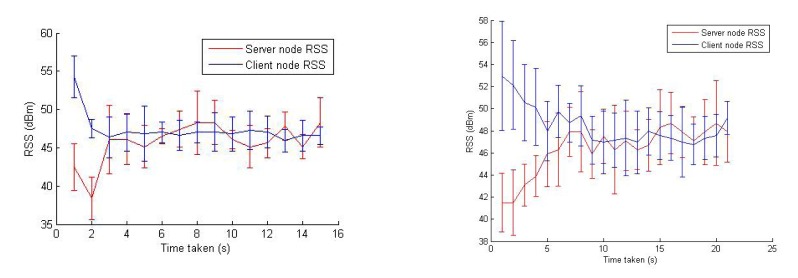
Change in the RSS values of server and client nodes at the relay node during the execution of the algorithm.

**Figure 18.**
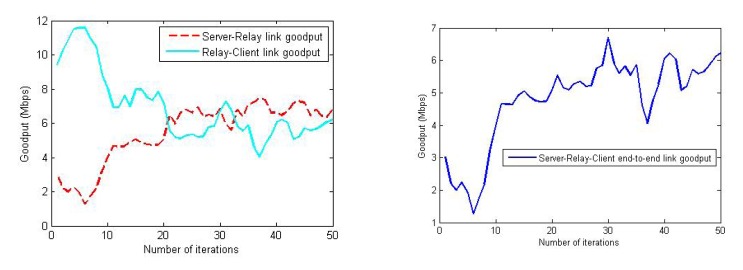
Change in the goodput values of server-relay, relay-client and end-to-end (server-client) links in one of the trials.

In Figure 17, two examples of the RSS balancing (and improvement) behavior are shown. The error bars are used to present the RSS values from various sensors on the relay node. It can be observed from this figure that the RSS is balanced quickly within the first few iterations, and then, the algorithm maintains the balance in the RSS between server and client nodes. Figure 18 plots the change in the goodput values during an experiment trial where the end-to-end link goodput (throughput) is consistently improved, even though the success score was zero (the relay node was trapped in a obstructed area). This confirms that the algorithm is able to achieve its objective of maximizing the network capacity, though sometimes, it cannot reach the global optimum position.

As a fundamental advantage of our proposed multi-sensor method is the redundancy (discussed in the Section 4.5.2), this method can be useful in environments with ionizing radiations that can affect the electronics in the nodes. This work is an initial step towards the goal of automatic adaptation of the wireless node to hostile environments.

## 7. Conclusions

In this paper, we have considered a novel method for improving the connectivity of mobile wireless robot nodes. Specifically, we have proposed a robust stochastic optimization (RSO) algorithm, using a stochastic gradient ascent (SGA) optimization method, for balancing and boosting the received signal strength (RSS) between two wireless nodes by optimizing the position of the relay node. Related work was referred to and compared with the proposed method throughout the development of the algorithm and its implementation. For the implementation of this algorithm, we devised a multi-sensor setup for the measurement of the radio signal strengths (RSS). The sensors are spatially distributed on the relay node, and we apply dedicated pre-processing techniques, such as an exponential moving average temporal filtering and an averaging spatial filtering. In principle, the proposed method can be implemented in any mobile robot platform. The effectiveness of the proposed solution for connectivity improvement was validated, both with simulations and field experiments in a CERN facility, using a mobile robot platform as the relay node and two stand-alone Raspberry Pi computers as the server and client nodes. Using the theoretical optimal solution as a measure of success, simulations and field experiments yielded a success rate of 88% and 78%, respectively. Although this leaves room for improvement, the algorithm considerably improved the RSS and the end-to-end network capacity in the non-successful cases nonetheless. It should be noted that the optimum positions in theory and practice might be different due to obstacles in the environment. Even though the proposed RSO algorithm cannot guarantee a global optimum position for the relay node due to physical (hardware) constraints and performance assessment limitations, it always guarantees improvement in the RSS and the network capacity, which are the main objectives in this research.

A fundamental advantage of the proposed solution is the redundancy in physical and algorithmic terms, which gives an increase in robustness. This robustness is needed in hostile environments where fail-safe operations of mobile robots are of supreme importance. The proposed methods are, however, not only intended to be deployed on ground wireless nodes (field robots), but also on unmanned aerial nodes, such as in quad-rotors.

Future work thus includes the implementation of the current developments in dynamic environments and improvement of the algorithm performance by integrating it with simultaneous localization and mapping (SLAM) algorithms for better obstacle detection and management. Extension to multi-tier architectures is also an avenue for future research.

## Figures and Tables

**Table 1.** Simulation results for all considered cases.

**Scenarios**	**MAE (m)**	**RMSE (m)**	**Distance Cost (m)**	**Time Cost (#iter)**	**Success Rate (%)**	**Speed of Convergence** $\left(\frac{m}{\#iter}\right)$
Case 1: LOS without noise	1.7	2.1	65.6	49.1	88	1.3
Case 2: LOS with 1 dBm noise	2.3	3.2	120.7	69.0	81	1.7
Case 2: LOS with 2 dBm noise	4.7	7.2	128.3	98.4	76	1.3
Case 3: NLOS with 1 dBm noise	3.3	6.1	161.8	81.9	78	1.9
Case 3: NLOS with 2 dBm noise	6.4	8.6	179.8	90.9	72	1.9
Case 3: deep-NLOS with 1 dBm noise	3.7	5.14	210.4	89.2	75	2.3
Case 3: deep-NLOS with 2 dBm noise	6.9	9.5	210.1	95.5	65	2.2

**Table 2.** Summarized results of the field trials.

	**Mean Value**	**Standard Deviation**
RSS improvement (dBm)	16.7	6.6
Success rate %	78	6.7
Time taken (s)	10.6	3.9
Distance traveled (m)	4.7	4.4
